# Using soil seed banks to assess temporal patterns of genetic variation in invasive plant populations

**DOI:** 10.1002/ece3.1043

**Published:** 2014-04-04

**Authors:** Mark Fennell, Tommy Gallagher, Luis Leon Vintro, Bruce Osborne

**Affiliations:** 1 University College Dublin, School of Biological and Environmental ScienceBelfield, Dublin 4, Ireland; 2 RPS GroupWillow Mere House, Compass Point Business Park, St Ives, Cambridgeshire, PE27 5JL, U.K.; 3 University College Dublin, School of PhysicsBelfield, Dublin 4, Ireland; 4 University College Dublin, School of Biological and Environmental ScienceBelfield, Dublin 4, Ireland

**Keywords:** AFLP, biological invasions, *Gunnera tinctoria*, population genetics, soil seed banks, stochastic evolutionary processes

## Abstract

Most research on the genetics of invasive plant species has focused on analyzing spatial differences among existing populations. Using a long-established *Gunnera tinctoria* population from Ireland, we evaluated the potential of using plants derived from seeds associated with different soil layers to track genetic variation through time. This species and site were chosen because (1) *G. tinctoria* produces a large and persistent seed bank; (2) it has been present in this locality, Sraheens, for ∼90 years; (3) the soil is largely undisturbed; and (4) the soil's age can be reliably determined radiometrically at different depths. Amplified fragment length polymorphic markers (AFLPs) were used to assess differences in the genetic structure of 75 individuals sampled from both the standing population and from four soil layers, which spanned 18 cm (estimated at ∼90 years based on ^210^Pb and ^137^Cs dating). While there are difficulties in interpreting such data, including accounting for the effects of selection, seed loss, and seed migration, a clear pattern of lower total allele counts, percentage polymorphic loci, and genetic diversity was observed in deeper soils. The greatest percentage increase in the measured genetic variables occurred prior to the shift from the lag to the exponential range expansion phases and may be of adaptive significance. These findings highlight that seed banks in areas with long-established invasive populations can contain valuable genetic information relating to invasion processes and as such, should not be overlooked.

## Introduction

Invasive non-native species are estimated to cost the world economy hundreds of billions of dollars every year (Pimentel 2011) and are considered one of the leading threats to ecosystem functioning and biodiversity (Vila et al. 2010). Understanding the processes involved in the establishment of non-native species and how they contribute to successful invasions is therefore important from both an economic and an ecological standpoint.

After initial colonization, a lag phase is frequently observed prior to the rapid range expansion of introduced plants (Mack 1985), and it has been proposed that evolutionary processes (stochastic and adaptive) that alter the genetic structure of a population during this period could contribute to invasive success (Lee 2002; Cox 2004; Prentis et al. 2008; Fennell et al. 2010). To date, research on invasive plant population genetics has focused on comparing genetic diversity between existing populations from native and introduced ranges (Suarez and Tsutsui 2008), and between populations at early and later stages of invasion (Chun et al. 2009; Fennell et al. 2010). Such studies have provided insights into the role that both adaptive and stochastic evolutionary processes play in shaping the genetic structure of introduced populations; however, they do not directly address alterations in genetic variation through time and have not always provided convincing evidence that evolutionary modifications have contributed to successful invasions.

Recently, Chun et al. (2010) used herbarium specimens to investigate historical alterations in the genetic structure of invasive *Ambrosia artemisiifolia* populations. The results suggested that current invasive populations have arisen from active gene flow and the subsequent admixture of historical populations, incorporating new alleles from multiple introductions. This study showed that including a temporal element in population genetic studies can provide useful information on the evolutionary processes involved in forming invasive populations. However, herbarium material of past populations that are amenable to genetic analyses is rarely available.

Soil seed banks play an important role in determining the structure of plant communities (Harper 1977; van der Valk and Davis 1978), providing a belowground reservoir of genetic variability (Templeton and Levin 1979; Baker 1989; Leck et al. 1989; Thompson et al. 1997; Honnay et al. 2008). A meta-analysis of studies that compared the genetic diversity contained within seed banks to that of the standing aboveground vegetation revealed generally higher allele counts in seed banks compared to the standing populations, but with comparable levels of heterozygosity and percentage polymorphic loci (Honnay et al. 2008). It was concluded that the difference in allele counts is primarily due to the presence of rare alleles in the seed bank, that aboveground vegetation is generally genetically similar to the seed bank, and that any genetic differences are likely to be the result of local selection. This indicates that the genetic structure of seed banks provides a good representation of the genetic structure of aboveground populations. However, research to date on the genetic structure of seed banks has been carried out on endemic species, not non-native invasive ones, and there has been little, if any, research that has used seed banks to investigate temporal variations in genetic structure of a non-native invasive population.

Since seeds in deeper soils are generally older (Thompson et al. 1997; Espinar et al. 2005), it is hypothesized that seed banks can provide the information required to examine genetic variation through time, thus providing insights into the evolutionary processes involved in forming invasive populations. To test this hypothesis, *Gunnera tinctoria* seeds were germinated from soil obtained at varying depths at a site in Sraheens, Ireland. *G. tinctoria* is a native of Chile, but has become invasive in a number of countries worldwide, including Ireland, the United Kingdom, the Azores, the USA, and New Zealand, and it produces a persistent seed bank (Osborne et al. 1991; Gioria and Osborne 2009). Further details on the biology and ecology of *G. tinctoria* can also be found in Gioria and Osborne (2013). Historical and other evidence indicates *G. tinctoria* has been present in the study area for more than 90 years (Fennell et al. 2012; Gioria and Osborne 2013), and the negative impacts of this species are likely to be exacerbated by climate change (Fennell et al. 2013). The soil at this location also possesses characteristics that would limit seed movement and decomposition, and the soil layers can be reliably dated.

Amplified fragment length polymorphic (AFLP) markers (Vos et al. 1995) were used to investigate the influence of stochastic processes (founder effects and gene flow) on variations in genetic structure between soil layers and plants growing aboveground. This information was combined with the age profile of the soil, determined using ^210^Pb and ^137^Cs dating, and compared to the recorded changes in distribution of the species at the corresponding time intervals. Potential issues that might influence the reliability of this approach, including how representative the genetic information is of former populations, seed movement between soil layers and variations in seed mortality, dormancy and viability, are also discussed.

## Materials and Methods

### Soil cores

**Figure 1:**
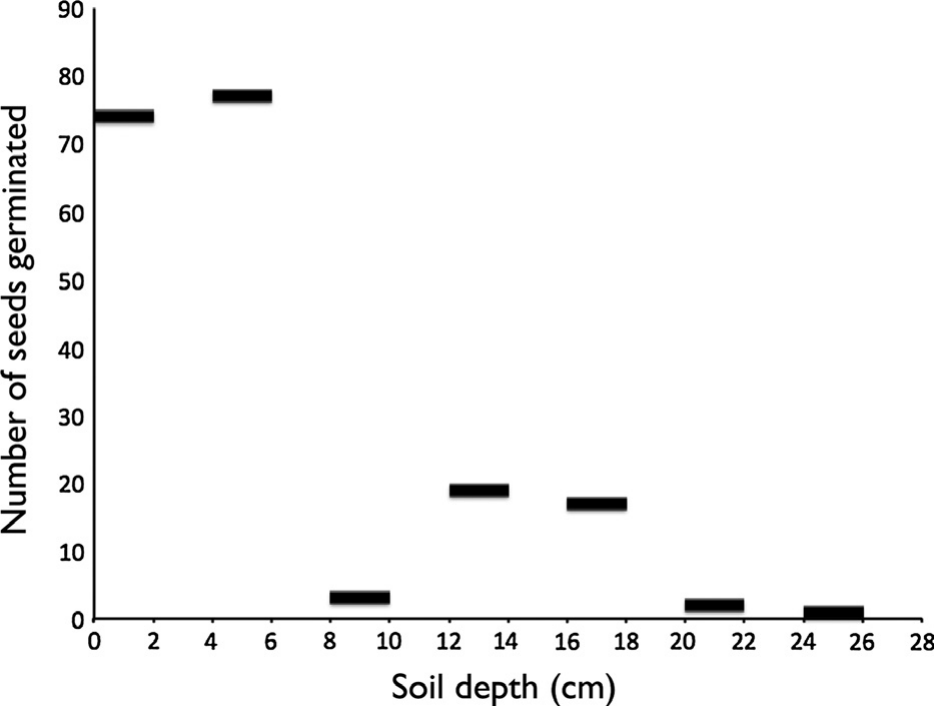
Total number of *Gunnera tinctoria* seeds germinated at different soil depths.

Three soil cores were extracted from a heavily invaded unimproved grassland area (80 × 70 m) at Sraheens, Co Mayo, Ireland, where *G. tinctoria* forms a largely monospecific stand (Longitude: 53.920769, Latitude: −9.947208). The soil in this area is a fine minerotrophic peaty gley (Hickey 2002) consisting of fine particles and with little earthworm activity, meaning that seed movement within this soil would likely be relatively low (Chambers et al. 1991). Based on pollen dating, the arrival of *G. tinctoria* at this site was estimated to be in the 1920s (Hickey 2002). The location of each soil core within the Sraheens site was chosen to maximize the distance between the samples (25 m minimum separation distance). Plastic piping with a diameter of 12.5 cm and a length of 35 cm was used to obtain soil cores, and these were driven into the soil up to their maximum length. The pipes containing the soil cores were then carefully removed from the ground and stored at 4°C within 24 h. Each soil core was then divided into 2-cm-thick slices. Soil from each layer was filtered through a sieve (4 mm diameter) to remove any fragments of rhizome or root that may have been present and then refrigerated at 4°C for up to a month. Potting trays (length: 52 cm, width: 42 cm, depth: 10 cm) were lined with vermiculite, and 2 cm of sand was added to the bottom of each tray to facilitate drainage. Slices from depths 0–2, 4–6, 8–10, 12–14, 16–18, 20–22, 24–26 cm, from each soil core, were mixed with compost (John Innes no. 2) and potted in the trays. The trays were grouped by soil core in a greenhouse and kept at ∼20°C under ambient light conditions for 3 months. Soil was periodically stirred using a glass rod to encourage seed germination; mixing of soil continued until no new germination was observed for a 30-day period. The number of seedlings that germinated from each soil layer is shown in Fig. 1.

### Plant material and sediment dating

Plant material was sampled from both the standing population and from seedlings that germinated from the soil cores. Five *G. tinctoria* plants that were growing adjacent to each soil core were sampled by removing approximately 5 cm^2^ of leaf material. Samples from aboveground were preserved on site by drying using silica gel (Environogel), following the method described by Chase and Hill (1991). Once the seedlings from the soil cores had reached a sufficient size (large enough to provide 0.06 g of dry leaf material), they were harvested and preserved using the same silica gel technique. Where five or more seedlings had germinated, five seedlings were randomly sampled from each soil depth and in each soil core, providing a total of fifteen seedlings per soil layer. Sufficient material was retrieved from soil core depths 0–2, 4–6, 12–14, and 16–18 cm.

The soil used for dating was collected prior to the present study, but from the same areas (within ∼20 m) as those used for the seed bank analysis (collected in 2010). One dating core was collected in 2002 and one in 2006. The cores, 50 cm long and 10 cm in diameter, were sealed with tape at both ends and transported back to the laboratory. After careful removal of soil from the cores, the outer ∼1 cm of soil was removed in case of contamination. The inner part of the core was then cut into 1-cm discs and air dried at approximately 35°C for at least a week. These samples were then passed through a 2-mm sieve to remove all large particles and stones. Subsamples (∼10 g) of the dried sections were then finely ground and homogenized before placing them in low background plastic counting vials prior to ^210^Pb and ^137^Cs dating. The age of the different soil depths was determined radiometrically by high-resolution gamma spectrometry using an Ortec-supplied n-type GMX Series Gamma-X HPGe coaxial photon detector (model GMX-15185) with a relative efficiency of 22.5% and a resolution of 0.546 keV and 1.69 keV (FWHM) at energies of 5.9 keV and 1.33 MeV, respectively. Energy and efficiency calibrations were established using a mixed radionuclide standard solution (SRM 4276) containing 154Eu and 155Eu, supplied by the National Institute of Standards and Technology (US), a mixed radionuclide standard solution (7081/4) containing ^241^Am, ^137^Cs, and ^60^Co, supplied by CERCA Framatone ANP (France), and a ^210^Pb standard solution (S6/19/110) supplied by Amersham International (UK). Self-absorption of low-energy γ-rays within the sample was corrected for after the method of Yang and Ambats (1982).

Total ^210^Pb activity concentrations were measured via its gamma ray emission at 46.5 keV, while ^226^Ra, used to establish “supported” ^210^Pb concentrations, was determined by assuming equilibrium with its daughter nuclides, ^214^Pb (gamma emission at 351 keV) and ^214^Bi (gamma emission at 609 keV), following an appropriate equilibration period of at least 3 weeks in a sealed container. Unsupported (excess) ^210^Pb was calculated as the difference between the measured total ^210^Pb and the estimated ^226^Ra content. Concentrations of ^137^Cs and ^241^Am were measured via their gamma emissions at 661.7 and 59.5 keV, respectively. Chronologies and sediment accumulation rates were established by applying the CIC and CRS hypotheses to the measured ^210^Pb excess data (Appleby and Olfield 1978).

Soil depths between 0 and 6 cm are referred to as shallow soil layers, and soil depths between 12 and 18 cm are referred to as deep soil layers. In total, 75 plants were sampled: 60 individual *G. tinctoria* plants from three soil cores across four soil depths, as well as 15 plants from the standing population.

### DNA isolation and AFLP procedures

Isolation of DNA was performed following the protocol outlined by Pich and Schubert (1993). AFLP procedures (Vos et al. 1995) were performed following the protocol described by Liscum (1999) with only minor modifications, as described by Fennell et al. (2010). Four primer pair combinations were used, and 182 putative loci were scored.

### Data analysis

From the 75 samples, fragments between 250 and 500 bp were scored as present (1) or absent (0) using the band-matching component of GelCompar2 3.0 (Applied Math, Sint-Martens-Latem, Belgium) and transformed into a binary 1/0 character matrix showing the presence or absence of each fragment for each sample. Fragments with a band frequency below 5% were excluded from the analysis as they are generally considered to be artifacts (Rajagopal et al. 2009). Fragments smaller than 250 bp were almost entirely monomorphic and formed a dense pattern; therefore, they were also excluded from the analysis (Vekemans et al. 2002).

The total allele count (*N*_l_), the number of private alleles (*N*_p_), and the percentage of polymorphic alleles (P_pl_) were calculated for each soil layer within each soil core using GENALEX 6.2. Using the distance component of GENALEX 6.2, the level of genetic diversity for each soil layer within each soil core was calculated based on the Shannon index of diversity (I) (Shannon and Weaver 1949). The percentage increases in *N*_l_, P_pl_, and I relative to their values in the next deepest soil layer were calculated. The values for the individuals from the deepest soil layer (16–18 cm) were not included as there was no deeper soil layer for comparison.

Principal coordinate analysis (PCO) was performed using the PCO component of PAST 1.94b (Hammer et al. 2009) to visualize the grouping of samples into clusters. For the PCO analysis, principal components were extracted from a distance matrix produced using the Jaccard (1901) similarity coefficient for binary data. PCO scatter plots were computed showing the clustering of all individuals and the clustering of individuals from the soil cores only.

An analysis of molecular variation (AMOVA) was performed using GENALEX 6.2 (Peakall and Smouse 2006). Due to the dominant nature of AFLP markers, each presence/absence of a band was treated as a haploid allele with the second allele at each locus recorded as missing (Bonin et al. 2007), an assumption required by GENALEX 6.2 for the AMOVA and allele frequency analyses. The partitioning of molecular variation among soil layers (ϕ_PR_), and within soil layers (ϕ_PT_), was calculated using a squared Euclidean distance matrix (9999 permutations). AMOVA was also performed within each soil core so that differences between ϕ_PR_ and ϕ_PT_ within soil cores could be characterized and compared.

## Results

### Germination rates

A significant negative correlation (*R*^2^ = 0.67, *P* < 0.025) was identified between germination rates and soil depth, with fewer seeds germinating in soils obtained from deeper soil core layers (Fig. 1). Very few seeds germinated from soil depths below 20 cm (1–2 seeds/layer), and only three seeds germinated from the 8–10 cm soil depth; therefore, these layers were not included in the analysis.

### Soil dating

Based on ^210^Pb and ^137^Cs dating, the soil in this area was determined to have an average accretion rate of ∼0.20 cm·year^−1^, corresponding to 5 years·cm^−1^. Furthermore, the pattern (i.e., a smooth, unbroken descending line) of ^210^Pb and ^137^Cs distribution indicated that the soil was relatively undisturbed.

### Genetic variability analysis

An analysis of molecular variance (AMOVA) revealed that 72% (*P* < 0.0001) of the total genetic variation was partitioned within soil layers and 97% (*P* < 0.0001) of total genetic variation was partitioned within soil cores (data not shown). This confirms that the patterns of genetic variation observed are due to differences between layers and not between cores.

**Figure 2:**
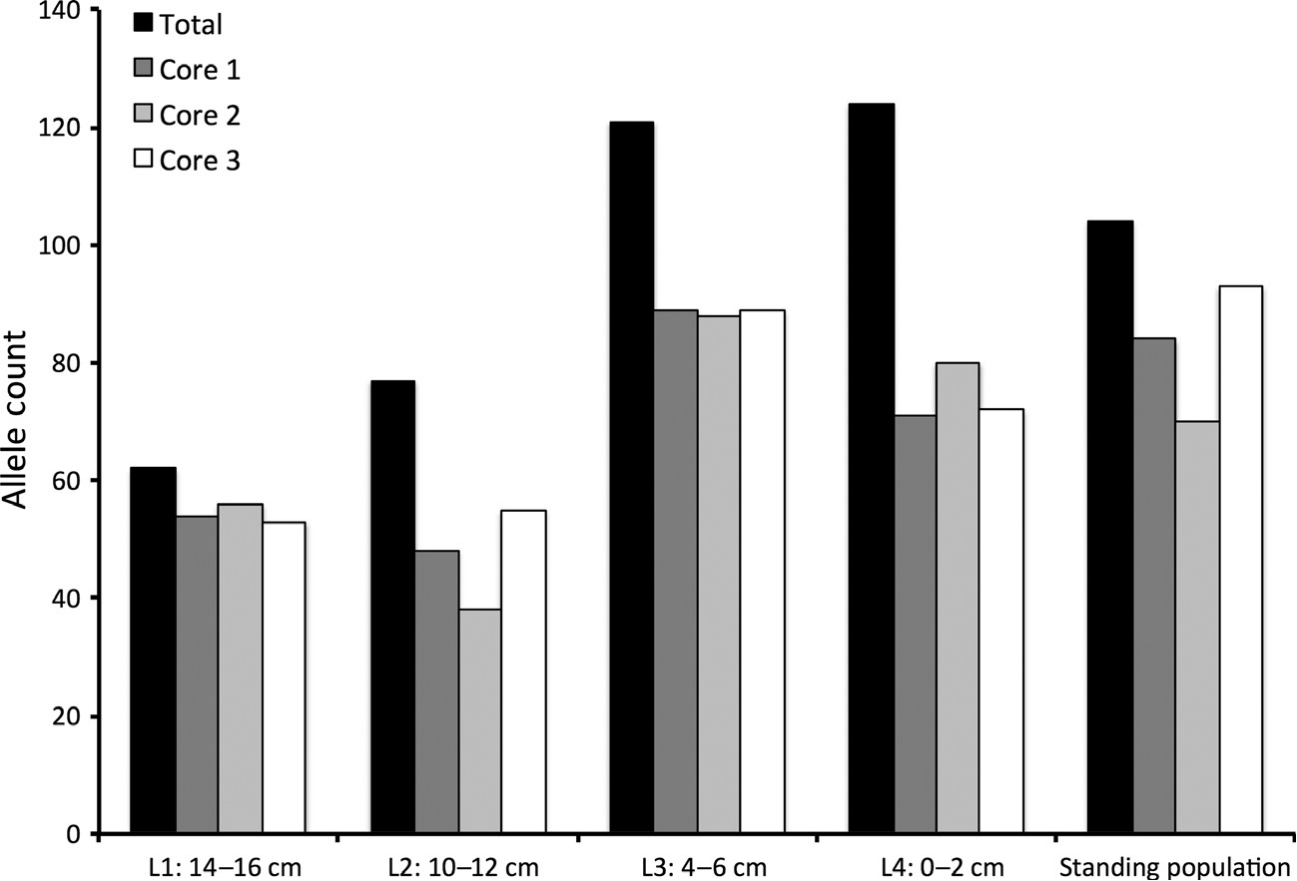
Total allele counts for each layer and for the different cores within each layer.

When data were aggregated for each soil layer, it was observed that seeds germinated from deeper layers had a lower total allele count (*N*_l_) (Table 1, Fig. 2), a lower percentage of polymorphic alleles (P_pl_) (Table 1), and a lower population genetic diversity (I) (Table 1). Increases in *N*_l,_ P_pl_, and I had a significant negative correlation with soil depth (respectively: *R*^2^ = 0.96, *P* < 0.018; *R*^2^ = 0.94, *P* < 0.029; *R*^2^ = 0.95, *P* < 0.022). For the standing population, *N*_l,_ P_pl_, and I were higher than those in deep soil, but lower than those in shallow soil (Table 1, Fig. 2). When soil layers were analyzed independently for each soil core, the correlation between *N*_l,_ P_pl_, and I and soil depth was not as strong, but remained significant (respectively: *R*^2^ = 0.58, *P* < 0.004; *R*^2^ = 0.55, *P* < 0.005; *R*^2^ = 0.55, *P* < 0.006).

**Table 1:** *Gunnera tinctoria* samples from different soil layers/cores and from different soil layers within each soil core. The table includes assigned symbol (Sym), soil depth, estimated soil age at each depth, number of samples analyzed (*n*), total allele count (*N*_l_), percentage polymorphic alleles (P_pl_), and Shannon's diversity index (I ±SE)

Sample	Sym	Depth (cm)	Soil date	*n*	*N*_l_	*N*_p_	P_pl_ (%)	I (±SE)
Layer 1	L1	0–2	1923–1933	15	62	0	23.08	0.121 (0.017)
Core 1	L1-1			5	54	0	13.19	0.075 (0.014)
Core 2	L1-2			5	56	0	10.44	0.014 (0.013)
Core 3	L1-3			5	53	0	11.44	0.040 (0.014)
Layer 2	L2	4–6	1942–1952	15	77	2	40.66	0.228 (0.022)
Core 1	L2-1			5	48	1	17.03	0.106 (0.018)
Core 2	L2-2			5	38	0	11.54	0.066 (0.014)
Core 3	L2-3			5	55	0	15.38	0.087 (0.015)
Layer 3	L3	12–14	1980–1990	15	121	6	64.29	0.356 (0.021)
Core 1	L-1			5	89	1	32.97	0.192 (0.021)
Core 2	L3-2			5	88	0	24.73	0.158 (0.021)
Core 3	L3-3			5	89	1	32.42	0.191 (0.021)
Layer 4	L4	16–18	1999–2009	15	124	2	65.38	0.377 (0.022)
Core 1	L4-1			5	71	0	23.08	0.128 (0.018)
Core 2	L4-2			5	80	0	22.53	0.130 (0.018)
Core 3	L4-3			5	72	1	19.23	0.106 (0.016)
Standing	L5	NA	NA	15	104	9	56.04	0.314 (0.022)
Core 1	L5-1			5	84	2	41.76	0.245 (0.022)
Core 2	L5-2			5	70	0	31.32	0.189 (0.021)
Core 3	L5-3			5	93	0	42.86	0.246 (0.021)

For the aggregated data, increases in *N*_l_ correlated linearly with increases in the percentage of polymorphic alleles (P_pl_) and population genetic diversity (I) (*R*^2^ = 0.97, *P* < 0.013 and *R*^2^ = 0.96, *P* < 0.016, respectively). There was a strong linear relationship between I and P_pl_ (*R*^2^ = 0.99, *P* < 0.001).

Private allele counts (*N*_p_) fluctuated between 0 and 9 (Table 1) and showed a general upward trend as depth decreased; however, the linear correlation between depth and private allele count was not significant (*R*^2^ = 0.52, *P* < 0.167).

**Figure 3:**
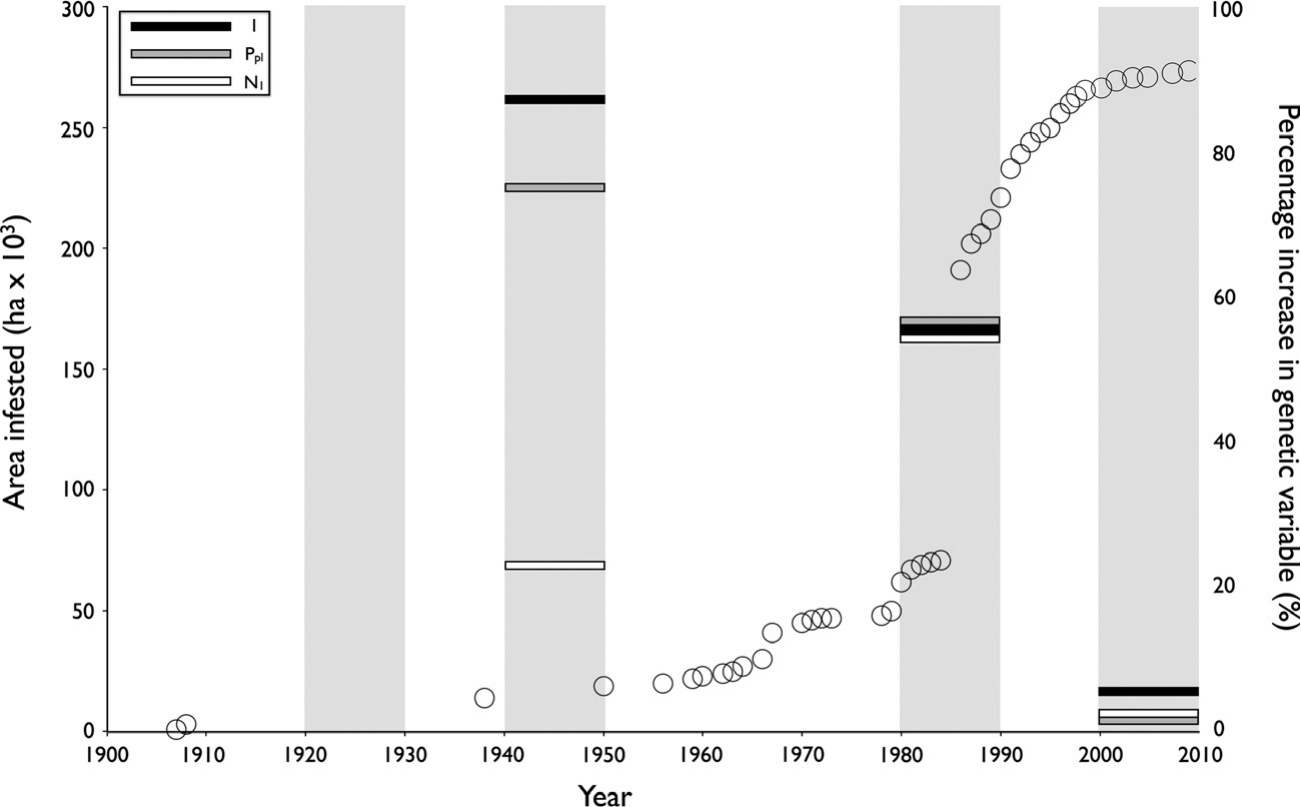
Figure showing the percentage increase in I, P_pl_, and *N*_l_ relative to individuals from the next deepest soil layer (horizontal bars). Analyzed soil layers are shown in grey. Open circles represent the area infested by *Gunnera tinctoria* versus time, based on empirical records (Preston et al. 2002).

The largest percentage increase in P_pl_ and I, relative to individuals from the next deepest soil layer, occurred in plants germinated from the 12–14 cm (L3) soil layer (Fig. 3). When these were plotted against the rate of population expansion for *G. tinctoria*, gained from distribution records (Preston et al. 2002), the greatest change was observed to have occurred during the transition from the lag to the exponential range expansion phases. The largest percentage increase in *N*_l_, relative to individuals from the next deepest soil layer, occurred in plants germinated from the 4–6 cm (L2) soil layer (Fig. 3).

**Figure 4:**
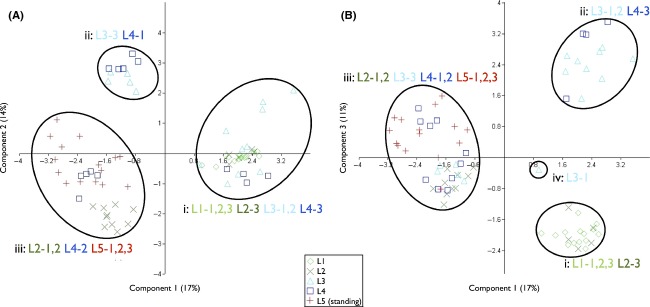
PCO analysis of the AFLP data set for *Gunnera tinctoria* samples from all soil layers and the standing population. The main clusters are circled with the origin layer (L) of the clustered seeds noted. (A) PCO analysis based on principal components 1 and 2. (B) PCO analysis based on principal components 1 and 3.

A principle coordinate analysis (PCO) of the presence/absence allelic data grouped the samples into three clusters (Fig. 4). Principal components 1, 2, and 3 accounted for 42% of the variation within the data set. The PCO analysis based on principal components 1 and 2 grouped the samples into three clusters (Fig. 4(A: i, ii, and iii)). The majority of samples (20 of 30) from deep soil layers (L1-1,2,3, and L2-3) formed a cluster with half of the samples (15 of 30) from shallow soil layers (L3-1,2, and L4-3) (Fig. 4(B: i)). The majority of the other samples (10 of 15) from shallow soil layers (L3-3 and L4-1) formed a distinct cluster (Fig. 4(A: ii)). The remaining samples were contained within another large cluster (Fig. 4(A: iii)). This cluster contained all samples from the standing population, the final five samples from the shallow soil layers (L4-2), and the remaining ten samples from the deep soil layers (L2-1,2). The PCO analysis based on principal components 1 and 3 grouped the samples into three main clusters (Fig. 4(B: i, ii, and iii)) and one outlier (Fig. 4(B: iv)). The majority of samples (20 of 30) from deep soil layers (L1-1,2,3, and L2-3) formed a distinct cluster (Fig. 4(B: i)). Half of the samples (15 of 30) from shallow soil layers (L3-1,2 and L4-3) formed another cluster (Fig. 4(B: ii)). Most other samples were contained within another large cluster (Fig. 4(B: iii)). This cluster contained all samples from the standing population, most of the other half of the samples (14 of 15) from the shallow soil layers (L3-3 and L4-1,2), and the remaining samples (10 of 30) from the deep soil layers (L2-1,2). In almost all cases, samples from the same layer in the same core clustered together.

## Discussion

To test the feasibility of using plant material derived from seed bank soil cores to track changes in population genetic structure through time, this study investigated differences in the pattern of genetic variation between *G. tinctoria* seeds germinated from different soil depths (dated using ^210^Pb and ^137^Cs analysis) and compared them with plants from a standing population. There was a general pattern of increasing genetic diversity in the seed bank with time, with the greatest increase relative to deeper soils occurring before the transition between lag and expansion phases. Germination rates were lower in seeds recovered from deeper soils, and total allele counts (*N*_l_), percentage polymorphic alleles (P_PL_), and the level of genetic diversity (I) decreased with increasing depth; however, based on PCO analysis, there were no clear population structure differences between the different layers. Four main nonmutually exclusive influences, which could have contributed to the observed allelic patterns, have been identified. These influences are as follows: differences in seed mortality/viability at different depths; seed movement through the soil; sample size; environmental conditions in the growth environment; errors determining soil accretion rates; and evolutionary processes (selection, founder effect, gene flow, etc.). The evidence indicates that evolutionary processes play the most significant role in influencing the observed patterns. To explain how this conclusion was reached, and to assess the role of other factors, the various possibilities are considered sequentially and evaluated against the available evidence.

### The influence of seed viability

The mechanisms that govern seed viability are not fully understood; however, seed longevity is believed to be influenced by a combination of genetic, physiological, and environmental factors (Fenner and Thompson 2005). The length of time that seeds can remain viable in soil is highly variable (Thompson et al. 1997), ranging from less than a year to over one thousand years in extremely rare cases (Thompson et al. 1997).

To our knowledge, there has been no direct assessment of *G. tinctoria* seed longevity. The closest related species for which seed longevity estimates are available is *Gunnera magellanica*. Based on burial and exhumation experiments (Arroyo et al. 2004), seeds that had not germinated after 755 days' burial remained highly viable (78.5% viability). It was also concluded that *G*. *magellanica* has the potential to produce persistent seed banks. The Gunneraceae also share some characteristics with the Fabaceae (González and Bello 2009), which have frequently been observed to have seeds that remain viable for 50 years or more (Becquerel 1907; Turner 1933). However, members of the same genus do not always share seed viability characteristics. For example, once exhumed, *Verbascum blattarai* had 42% viable seeds after 100 years of burial, whereas for *Verbascum thapsus,* only 2% of seeds were viable (Kivilaan and Bandurshi 1981).

We must, therefore, look at less direct sources of evidence to evaluate the potential longevity of *G. tinctoria* seeds. *G. tinctoria* seeds are very small and spherical (1–1.5 mm diameter) (Williams et al. 2005; Gioria and Osborne 2013). It has been observed that small-seeded families generally have the highest proportion of long-term seed persistence records (Thompson et al. 1997), and small, nearly spherical seeds tend to be the most persistent of all (Bakker et al. 1996; Schwienbacher et al. 2010). Viable *G. tinctoria* seeds have been found buried deep in soils, down to 20 cm, at many locations with different soil types and conditions (Fig. 1; see also Hickey 2002). Based on ^210^Pb and ^137^Cs dating, the soil at these depths has been estimated to be nearly a century old and consistent with records for the earliest occurrence of naturalized material (Gioria and Osborne 2013). A previous study carried out in the same area showed that *G. tinctoria* seeds found in deep soils are highly viable (up to 68% viability) (Hickey 2002). The patterns of ^210^Pb and ^137^Cs distribution in soils from Sraheens also indicate that the soil is largely undisturbed. Because seed movement in soil is slow (Bekker et al. 1998), it is generally true that deeper seeds are older, except in severely disturbed soils (Harper 1977; Leck et al. 1989; Thompson et al. 1997; Espinar et al. 2005). There is also a strong correlation between species where viable seeds are found at some depth in the soil and extended seed longevity (Baker 1989; Thompson et al. 1997; Bekker et al. 1998). *G. tinctoria* also produces large persistent seed banks (Gioria and Osborne 2009), which would not be possible if its seeds were not relatively long-lived.

In addition, the soil conditions of the Sraheens site could contribute to the longevity of seeds. More stable temperatures and moisture levels in deeper soils can help maintain seed viability (Fenner and Thompson 2005). The soil at Sraheens has a low mean annual temperature (∼10**°**C) and, due to its high water content, would be low in oxygen (Bhattarai et al. 2005). Low temperatures and reduced oxygen concentration can reduce microbial activity in the soil (Bhattarai et al. 2005) and increase seed longevity. Low temperatures and limited access to oxygen are also used to ensure low metabolic activity and delay seed aging in the Svalbard Global Seed Vault. Based on this evidence, it is probable that *G. tinctoria* produces seeds that can remain viable in the fine minerotrophic peaty gley soil at Sraheens for many decades.

It could be argued that higher levels of seed loss, due to either enhanced mortality or reduced viability in deeper soil, influenced the values obtained for *N*_l_, P_PL_, and I in deep soil layers. However, in order for depth-related changes in seed mortality or seed viability to have a meaningful influence on the results, these changes would have to be nonrandom in nature. Given that seeds from deeper soil layers are almost certainly older, they are likely to have experienced higher rates of seed mortality/decreased seed viability than seeds in shallow cores (an assumption backed up by the negative relationship observed between germination rates and soil depth). However, unless this loss of seeds had a genetic basis, that is, certain genotypes had an enhanced viability, selecting those individuals that remained in the soil for genetic analysis would be no different than randomly selecting the same number of individuals from the original intact seed population. Furthermore, as AFLPs are neutral markers, the genetic variation observed in this study would not be linked to the fitness of the organism and, therefore, would not be affected by natural selection (Merilä and Crnokrak 2001; McKay and Latta 2002). Fitness-related losses in seeds, therefore, should not have had a major impact on the results.

### The impact of the movement of seeds in soil

It could also be argued that the observed allelic patterns were influenced by the effect of seed movement between the soil layers. If we assume that the original seed population from the deeper soil layers was lost, then the pattern of decreasing germination rates with increasing depth could be explained by the downward migration of seeds from the shallower soil layers. However, if this was the case, then there would not be a large difference in the values of *N*_l_, P_PL_, and I observed between soil layers. This is because, although fewer seeds would have migrated all the way down to the deeper soil layers, the seeds that did reach those layers would be a random sampling of those from the shallower soil layers and, therefore, would be genetically similar to a random sampling of seeds exhumed from the larger seed population in the shallow soil layers. It can be assumed that some movement of soil/seeds has occurred over the past 90 years, largely due to the action of earthworms (Willems and Huijsmans 1994), although visual observations suggest that their numbers are small at this location. This may reduce the usefulness of soil age to conclusively determine the exact age of the seeds; however, it can still be argued that, overall, deeper seeds are older. Where seeds have been sampled from layers that are widely separated, as in this study, they are likely to be representative of seeds originating from populations that are temporally separated. Based on the fact that the soil in Sraheens is largely undisturbed, and on the differences observed for *N*_l_, P_PL_, and I between deep and shallow soil layers, disturbance of soil does not appear to have caused enough admixture of seeds from different layers to have a large effect on the results of this study.

On the other hand, based on the PCO analysis, the lack of clear population structure differences between layers, and in some cases the clustering together of individuals from different soil layers, could indicate that there has been some admixture of the soil. If the soil had undergone high levels of admixture, the difference between values of *N*_l_, P_PL_, and I for shallow and deep soil layers would require an external experimental error-based explanation, as it would appear that seed movement and seed loss have not had a large effect on the results.

### The impact of sample size and variable growth conditions

We have identified two main possible experimental errors, namely sample size and nonuniform environmental conditions in the greenhouse where the seeds were germinated.

First, the sample sizes used may not have been sufficient. Fifteen individuals, across three soil cores, were selected from each layer and from the standing population. This number was used because, due to lower germination rates in deeper layers, it was the maximum number that could be harvested from all layers. Brown (1989) and Brown and Biggs (1991) proposed that sampling 10% of the individuals in a population would capture 60–70% of the alleles present. The maximum number of individuals that germinated from any layer was 77 from which fifteen (∼20%) were sampled. However, this may not represent 20% of the total population, as some viable seeds may not have germinated and the three soil cores may not have captured a large enough cross-section of the total seed bank. Therefore, experimental error cannot be completely ruled out. However, compared with the sample sizes used in many other population genetic studies, the use of fifteen individuals to evaluate the genetic structure of a single small population is not unusual (Amsellem et al. 2000; Wen-Kun et al. 2007; Chun et al. 2009 and, 2010). Nevertheless, more soil cores per population and higher sample numbers would be beneficial in future studies.

Secondly, samples were grouped by soil core in the greenhouse, and the growth conditions may not have been uniform for all samples. These variables included shade patterns, and different proximities to ventilation and water sources for the different growth trays. Also, germination was not synchronous for all trays, instead occurring over several months. Temperature and light quality would have varied across this time period. These variables could have exerted, by chance, different selective pressures on different sets of trays. However, once again, due to the use of neutral genetic markers, selection should not have had a major influence on the allelic patterns observed. However, any possible environmental issues in the greenhouse would be minor as it was a semi-controlled environment. Regardless of future studies, the position of each sample should be assigned randomly.

### The effect of errors in the determination of accretion rate and soil age in different cores

The lack of definitive population structure differences between soil layers could be explained if the ages of analogous soil layers in different cores were not uniform. The soil dates used in this study are from averages obtained from data originating from cores collected on two previous studies. The accretion rates obtained (0.204 cm·year^−1^ (SD = 0.109)) were variable. If the soil depths in the different cores did not correspond to the same dates, then we would not expect to see definitive population structure differences between layers. However, samples from sequentially deeper layers from each individual core would still have been sequentially older, and changes through time could still have been observed for *N*_l_, P_PL_, and I. In future, however, soil age profiles should preferably be obtained for each individual core and be examined independently.

### Combined analysis

On the basis of this assessment of the possible confounding factors associated with estimates of genetic differences in soil seed populations at varying depths, it is unlikely that seed loss, seed migration, and experimental error can explain the depth-dependent changes in *N*_l_, P_PL_, and I. It was hypothesized in a previous spatial analysis carried out in this area (Fennell et al. 2010) that gene flow played a major role in shaping the genetic structure of invasive populations. The study concluded that gene flow helped to counterbalance the reduction in population genetic diversity caused by founder effects and genetic drift, and potentially contributed to the populations' invasive success. The putative source population in this area had values of 97, 46%, and 0.229 for *N*_l_, P_PL_, and I, respectively. In this study, the low values observed for *N*_l_, P_PL_, and I, in the deepest soil layer (62, 23% and 0.121, respectively), the subsequent increases of the values with decreasing depth, and the higher levels observed in the shallowest soil layer (124, 65% and 0.377, respectively) would be indicative of a population that had previously undergone genetic bottlenecking and subsequently experienced gene flow influenced increases in allelic content through time. The higher number of private alleles in the standing population and the trend of increasing private allele counts with decreasing depth would also be indicative of new alleles arriving through time. Finally, from the PCO analysis, individuals from the deepest soil layer were distinct from the standing population. The results from this study, therefore, support the findings of Fennell et al. (2010).

An examination of the relationship between percentage changes in genetic variables and the invasion curve for *G. tinctoria* indicates that increases in genetic diversity may be associated with a shift from the lag to the exponential growth expansion phase. Although, owing to the use of neutral genetic markers, no specific inferences can be made, this could imply that adaptive evolution, benefitting from a larger gene pool, has played a role in the expansion of this introduced species.

The general utility of the approach used in this study is not limited to AFLP analysis. If such an approach was combined with an analysis of epigenetic variation, MS-AFLP (Hazen et al. 2002), together with an analysis of gene expression and transcriptome sequencing (Fox et al. 2013), additional information on the genetic contribution to invasive success could be acquired. Such information would make this an even more powerful tool for understanding links between genetic changes, and the establishment and range expansion of introduced plant species.

## Conclusion

The use of seedlings germinated from sequentially deeper and datable soil layers can be used to track changes in the population genetic structure of an invasive species through time. The success of this approach is likely to depend on the chosen species and location. A species with highly persistent seeds is required, as is a location with soil that has the appropriate characteristics and that can be reliably dated. Neutral genetic markers can be used effectively. An equal number of samples should be randomly selected from all soil layers.

Changes in the pattern of variation in *N*_l_, P_PL_, and I between deep soil, shallow soil, and the standing population indicate that existing invasive populations of *G. tinctoria* have arisen from an initial genetic bottleneck, followed by active gene flow from other locally occurring populations. As a result, the population allelic content has increased over time. This increase in genetic diversity may have counterbalanced any potentially fitness-reducing effects of genetic bottlenecking, thereby contributing to range expansion and, ultimately, invasive success.

If this approach is adopted and used to examine temporal changes in the population genetic structure of many invasive species across many environments, then invaluable insight could be gained into the genetic processes that lead to the success of invasive populations.
